# Predictors of post-operative mortality following treatment for non-ruptured abdominal aortic aneurysm

**DOI:** 10.1186/1468-6708-6-14

**Published:** 2005-09-07

**Authors:** Sigitas Urbonavicius, Henrik Vorum, Grazina Urbonaviciene, Mindaugas Trumpickas, Dainius Pavalkis, Bent Honoré

**Affiliations:** 1 Kaunas University of Medicine, Institute of Cardiology, Lithuania; 2 University of Aarhus, Institute of Medical Biochemistry, Denmark; 3 Kaunas University of Medicine, Clinic of Surgery, Lithuania; 4 Kaunas University of Medicine, Clinic of Cardiovascular Surgery, Department of Vascular Surgery, Lithuania

**Keywords:** abdominal aortic aneurysm, surgical treatment, mortality rate

## Abstract

The aim of this prospective study of patients undergoing repair of non-ruptured abdominal aortic aneurysm between 1999 and 2003 was to evaluate and compare risk factors for mortality after surgery, to determine a complex of informative factors for lethal outcome, and to define patient risk groups. Logistic regression analysis revealed a complex of informative factors, including female gender, previous myocardial infarction, age greater than 75 years, and clinical course of abdominal aortic aneurysm as important indicators for lethal outcome. A *risk score model *identified low-, moderate- and high-risk groups with mortality rates of 2.9%, 8.0% and 44.4%, respectively.

## Introduction

Care of patients with abdominal aortic aneurysm (AAA) has been a benchmark of progress in vascular surgery for more than 50 years. By the early 1990s, elective repair of AAA was regularly carried out with a mortality rate of less than 5% [1]. Risk of rupture increases rapidly after the AAA reaches 5 cm in maximal diameter. Most surgeons therefore recommend surgical repair of AAAs 5 cm in diameter or larger and conservative treatment of smaller AAAs. However, rupture sometimes occurs with AAAs less than 5 cm in diameter, whereas some AAAs that are 5 cm or larger in diameter never rupture [2].

The aim of this study was threefold: (1) to evaluate and compare risk factors that influence the outcome of treatment in patients undergoing surgery for non-ruptured AAA, (2) to identify a complex of informative factors for lethal outcome, and (3) to define various risk groups.

## Materials and methods

### Patients

From January 1999 to August 2003 100 patients with a diagnosis of AAA were treated surgically at the Department of Vascular Surgery at the Kaunas University of Medicine. Thirty-one patients (31%) had surgery for a ruptured AAA and were excluded from our study. The 69 remaining patients (69%) underwent surgery for a non-ruptured AAA; these cases were analyzed in our study. There were 56 bifurcated (37.7% biiliacal, 29% bifemoral, and 14.5% femoral-iliac) and 13 (18.8%) straight grafts. All patients analyzed in this study had a midline laparotomy incision, and during AAA resection, the abdominal aorta was clamped below the renal arteries. Sixty-one patients survived while eight patients (11.6%) died within 30 days after surgery. Variables recorded for each patient were categorized as preoperative, intraoperative or postoperative.

### Preoperative variables

#### Cardiac function

All patients had consultations with an internist and a cardiologist. Cardiac ultrasonography was performed, and left ventricular ejection fraction (LVEF) was estimated. Patients without coronary artery disease (CAD) or with minor CAD included asymptomatic patients, patients with normal electrocardiogram (ECG) results, and patients in whom myocardial infarction (MI) was diagnosed with electrocardiography more than 6 months earlier.

#### Renal system

Patients were considered as having normal or only minor renal dysfunction when the serum creatinine level was less than 110 μmol/L.

#### Respiratory system

Patients with normal pulmonary function included asymptomatic patients with normal results on chest radiographs and normal results on respiratory function assessments.

#### Abdominal aorta

AAA diameter was measured during examination of the patient with computerized tomography (CT) and ultrasonography. Forty-two patients (61%) were examined with aortography.

### Intraopeartive variables

Operative risk was assessed according to the American Society of Anesthesiologists (ASA) classification. The following variables were recorded: type of operation, abdominal aorta "cross-clamping" time, declamping hypotension, blood loss during operation, urine output during operation (ml per hour) and total operation time.

### Postoperative variables

We recorded the following variables: durability of artificial pulmonary ventilation, length of stay in intensive care, length of hospital stay after operation, and total length of hospital stay. Complications during the postoperative period were not analyzed in this study.

### Statistical analysis

All variables defined above were subjected to univariate analysis using Microsoft Excel 2000 to test their influence on the mortality. Fisher's exact test was performed with SPSS 10.0 for Windows and Statistics v.5 for Windows. The complex influence of several predictors on the probability of lethal outcome (LO) was evaluated using the multidimensional logistic regression model [3]. The significance of variables in the logistic model was evaluated using the likelihood ratio (G^2^) and Wald statistics. When using the multidimensional logistic model coefficients, we developed a risk score model for the evaluation of complexes of risk factors. The accuracy of significant variables for prediction of LO was estimated by means of Receiver Operating Characteristic (ROC) curves. Variables that were statistically significant (p < 0.05) between groups of survivors and deceased were entered into a maximum probability logistic regression program. For each significant variable in the logistic regression analysis, the odds ratio (OR) was calculated (95% confidence interval). In order to determine low-, medium-, and high risk groups, we used LO probability estimation in the multinomial regression model, or logit function of the probability estimation log (p/(1-p)).

## Results

Sixty-nine patients underwent surgery for non-ruptured AAA. For each patient a set of variables were compared between the surviving group of patients and the group of deceased. The variables that differed significantly between the groups of survivors and deceased were age, blood loss, days of hospitalization, days of hospitalization after operation and LVEF as given in Table 1. Variables like aneurysm size, diuresis, duration of operation and aortic cross clamping time did not differ significantly between the groups (not shown).

**Table 1:** Characteristics of patients undergoing surgical repair of non-ruptured abdominal aortic aneurysm (95% confidence interval of mean)

Variable	Survivors	Deceased	p
	n = 61	n = 8	
Age (years)	[69.0; 73.2]	[73.2; 85.7]	0.02
Blood loss (ml)	[1442; 1908]	[310; 6364]	0.01
Length of hospitalization (days)	[17.5; 23.1]	[2.7; 7.27]	0.006
Length of hospitalization after operation (days)	[11.3; 15.9]	[0.18; 3.2]	0.007
LVEF (%)	[43.4; 47.7]	[30.9; 45.0]	0.019

LVEF-left ventricular ejection fraction

Table 2 shows additional groups of binominal variables that differed significantly with respect to mortality rate. We found significantly higher mortality rate among patients above 75 years, among females, as well as among patients with previous MI, with symptomatic course of AAA, with insufficient respiratory function or with insufficient renal function. In our material the mortality rate was not significantly higher among patients with ischemic heart disease, arterial hypertension, aorto coronary bypass operation, diabetes mellitus or LVEF below 40% (not shown).

**Table 2:** Binominal variables among 69 patients operated for non-ruptured abdominal aortic aneurysm.

**Variable**	Number of operated patients	Number of deceased patients (%)	P (Fisher)
Age			
≤75 yrs.	51	3 (5.9%)	
>75 yrs.	18	5 (38.5%)	0.024
			
Distribution by sex:			
Males	52	5 (9.6%)	
Females	17	3 (17.6%)	0.008
			
MI in anamnesis			
Yes	34	7 (20.6%)	
No	35	1 (2.9%)	0.025
			
Clinical course of AAA			
Asymptomatic	48	3 (6.3%)	
Symptomatic	21	5 (23.8%)	0.04
			
Insufficient respiratory function			
Yes	18	5 (27.8%)	
No	51	3 (5.9%)	0.024
			
Insufficient renal function			
Yes	11	3 (27.2%)	
No	58	5 (8.6%)	0.01

MI-myocardial infarction

The ability of significant variables to predict LO was estimated by means of ROC curves. The area under the ROC curve is a measure of how well the groups are separated. The ROC curve's position above the mean line demonstrates the capability of the method to predict lethal outcome with some degree of precision. An area of 1 represents a perfect test. In our study, the area under the age curve was 0.778; CI [0.616; 0.94]; p = 0.011; the area under the sex distribution curve was 0.714; CI [0.5; 0.92]; p = 0.050; and the area under previous MI was 0.716; CI [0.55; 0.883]; p = 0.048 (not shown). Other variables were not found to be significant as estimated by the ROC curve.

To evaluate the informative parameters for LO, we used logistic regression. Age, previous MI, insufficiency of respiratory function, blood loss, LVEF and clinical course of AAA were confirmed as informative factors for LO (Table 3). Previous MI signified the highest risk; the value of this parameter was 8.8. The risk values associated with insufficiency of respiratory function and clinical course of AAA were somewhat lower (6.15 and 4.69, respectively). Each incremental blood loss of 100 ml during surgery was calculated to possess a risk value of 1.05. In our material the following variables were not considered as informative, size of diuresis, insufficiency of renal function and arterial hypertension.

**Table 3:** Prognostic values of the informative parameters for lethal outcome

Parameter	χ^2^	p	OR	CI
Age, years	7.36	0.006	1.14	[1.03–1.26]
Previous MI	5.85	0.016	8.8	[1.02–76.06]
Clinical course of AAA	4.01	0.045	4.69	[1.004–21.87]
Blood loss, ml	5.48	0.019	1.05	[0.99–1.11]
Insufficient respiratory function	5.42	0.02	6.15	[1.3–29.17]
LVEF, %	5.8	0.016	0.89	[0.81–0.99]

AAA – abdominal aortic aneurysm, OR – odds ratio, LVEF – left ventricular ejection fraction, CI – confidence intervals, MI – myocardial infarction.

A logistic model for LO was established by using an informative parameter method, taking into consideration the strong correlation between some of the variables. For this model, the optimal criterion was a p-value that was adequate for the logistical model criterion G^2^. The logistic regression model included the variables as long as the p-value was significantly decreasing. Two models turned out to be able to predict LO. One model included the variables patient gender, previous MI and age above 75 years:

p(LO) = 1/1+exp{1.83 × gender + 2 × previous MI + 1.44 × (age>75 years) - 6.5}

χ^2 ^= 14.5 p = 0.023

A second model included the variables patient gender, previous MI and clinical course of AAA:

p(LO) = 1/1+exp{1.8 × gender + 2.3 × previous MI + 1.12 × (clinical course of AAA) - 7.2}

χ^2 ^= 15.1 p = 0.0017

The risk score model allows evaluation of the groups for risk stratification. Score evaluation was performed by e^β^. The value of female gender (β = 1.83) was 6 (6.21); the value of previous MI (β = 2) was 7 (7.4); and the value of age greater than 75 years (β = 1.44) was 4 (4.23). The maximum sum of risk scores was 17. In 54 patients (78%), the score was less than 10, and mortality in this group was 3.7% (two patients). In 15 patients (22%), the score was higher than 10, and mortality in this group was 40% (six patients).

**Figure 1:**
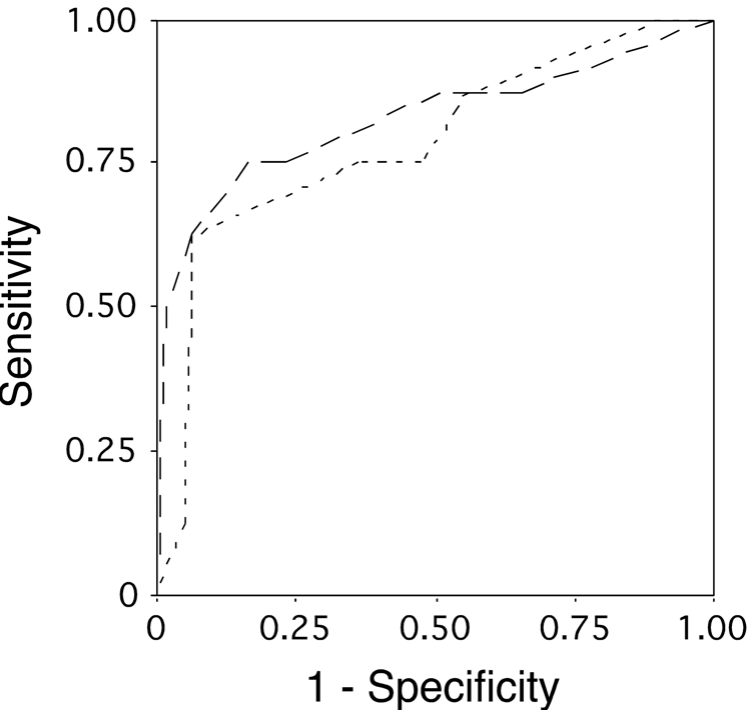
ROC curves of the logistic models: the 1st model (---) included gender, previous myocardial infarction and age above 75 years; the 2nd model (_ _ _ _) included gender, previous myocardial infarction and clinical course of abdominal aortic aneurysm.

Using the second logistic model, the value of female gender (β = 1.8) was 6 (6.1), the value of previous MI (β = 2.3) was 10 (9.94), and the value of clinical course of AAA (β = 1.12) was 3 (3.06). The maximal sum of scores was 19. In 35 patients (51%), the sum was less than 13, and mortality in this group was 2.9% (1 patient). Twenty-five patients (36%) had scores of 13 to 16, and two patients (8.0%) in this group died postoperatively. Nine patients (13%) had scores higher than 16; four patients (44.4%) from this group died postoperatively. These findings were used for establishing low-, moderate-, and high-risk groups (Table 4). The prognostic values of both models is given by means of ROC curves in Fig. 1. The area under the 1st logistic model curve was 0.78 (CI [0.6; 0.97]; p = 0.009) and the area under the 2nd logistic model curve was 0.83; (CI [0.63; 1.02]; p = 0.003). These data suggest that both models predict LO fair enough but the 2nd logistic model demonstrates a slightly better sensitivity.

**Table 4:** Risk groups according to the sums of scores in model 2

Risk groups	Score	Mortality (%)
Low-risk group	<13	2.9
Moderate-risk group	13–16	8.0
High-risk group	>16	44.4

## Discussion

The 30-day postoperative mortality rate reported in the literature ranges from 0 to 10.5%. In our material it is slightly higher, 11.6%, probably due to the small number of patients. In a previous report based on a large number of patients collected from studies published between 1985 and 1997, the mortality rate was 5.8% [4].

In the elective situation, advanced age and cardiac, pulmonary, and renal disease increase the risk of postoperative mortality [5]. As in previous studies, age, renal dysfunction, and previous MI were found to be strong independent predictors of postoperative death [6,7]. Recent data suggest that female gender may increase mortality associated with repair of AAA [7,8]. We found that age greater than 75 years, previous MI, clinical course of AAA, and female gender were informative factors for lethal outcome. Steyerberg and colleagues developed a clinical prediction model to estimate the operative mortality risk for individual patients undergoing elective AAA repair [9]. With respect to informative parameters, our logistic model was similar to that of Steyerberg. However, in our material we found that size of the aneurysm did not influence perioperative mortality.

Blood loss during the operation was a statistically significant factor for increased mortality. Other investigators have also concluded that blood loss during surgery is an informative factor for lethal outcome [10].

All postoperative complications increase length of stay in intensive care. However, postoperative complications were not analyzed in our study.

## Conclusion

Logistic regression analysis revealed a combination of informative factors for mortality after surgical repair of non-ruptured AAA. In one model these factors were female gender, previous MI, and age greater than 75 years. In a second model female gender, previous MI, and clinical course of AAA were important predictors of lethal outcome. The latter *risk score model *allows identification of groups of patients running a high risk for postoperative death. Mortality was 2.9% in the low-risk group, 8.0% in the moderate-risk group, and 44.4% in the high-risk group.
